# 4 in 1: Antibody‐free protocol for isolating the main hepatic cells from healthy and cirrhotic single rat livers

**DOI:** 10.1111/jcmm.13988

**Published:** 2018-11-12

**Authors:** Anabel Fernández‐Iglesias, Martí Ortega‐Ribera, Sergi Guixé‐Muntet, Jordi Gracia‐Sancho

**Affiliations:** ^1^ Liver Vascular Biology Research Group Barcelona Hepatic Hemodynamic Laboratory IDIBAPS Biomedical Research Institute Barcelona Spain; ^2^ Biomedical Research Networking Center in Hepatic and Digestive Diseases (CIBEREHD) Madrid Spain; ^3^ Hepatology Department of Biomedical Research Inselspital Bern University Bern Switzerland

**Keywords:** cirrhosis, hepatocytes, HSC, Kupffer cells, LSEC

## Abstract

Liver cells isolated from pre‐clinical models are essential tools for studying liver (patho)physiology, and also for screening new therapeutic options. We aimed at developing a new antibody‐free isolation method able to obtain the four main hepatic cell types (hepatocytes, liver sinusoidal endothelial cells [LSEC], hepatic macrophages [HMΦ] and hepatic stellate cells [HSC]) from a single rat liver. Control and cirrhotic (CCl_4_ and TAA) rat livers (n = 6) were perfused, digested with collagenase and mechanically disaggregated obtaining a multicellular suspension. Hepatocytes were purified by low revolution centrifugations while non‐parenchymal cells were subjected to differential centrifugation. Two different fractions were obtained: HSC and mixed LSEC + HMΦ. Further LSEC and HMΦ enrichment was achieved by selective adherence time to collagen‐coated substrates. Isolated cells showed high viability (80%‐95%) and purity (>95%) and were characterized as functional: hepatocytes synthetized albumin and urea, LSEC maintained endocytic capacity and in vivo fenestrae distribution, HMΦ increased expression of inflammatory markers in response to LPS and HSC were activated upon in vitro culture. The 4 in 1 protocol allows the simultaneous isolation of highly pure and functional hepatic cell sub‐populations from control or cirrhotic single livers without antibody selection.

## 1 INTRODUCTION

The liver is the largest internal organ in humans being the main site for macromolecule synthesis and storage, blood clearance and drug metabolism.^1^ Hepatocytes, the parenchymal fraction of the liver, approximately account for the 60% of the liver mass and are responsible for the detoxification, bile synthesis and storage functions of the organ.^2^ Hepatocytes alone are not competent to perform the abovementioned functions; they work as an integrated community with the so called non‐parenchymal cells (NPC), mainly: liver sinusoidal endothelial cells (LSEC), hepatic stellate cells (HSC) and resident hepatic macrophages (HMΦ) also known as Kupffer cells.^3^, ^4^

LSEC (19% of the liver mass) are highly specialized endothelial cells characterized by the presence of transcellular pores called fenestrae and the lack of basal membrane.^5^, ^6^ This unique cell type not only lines the physical barrier between blood and hepatocytes but is also involved in regulating sinusoidal blood flow, tissue homeostasis, immune response and macromolecular waste clearance.^7^, ^8^ HMΦ (10%) represent the resident tissue macrophages, which upon liver damage synthesize and secrete immune modulators.^9^, ^10^ Finally, HSC approximately account for the 6% of the liver mass and are found in the space of Disse, surrounding LSEC. Major roles of HSC are regulation of the vascular tone and retinoid storage. Besides, upon liver injury, HSC activate and acquire a myofibroblast‐like phenotype in which extracellular matrix (ECM) is actively produced and deposed in order to limit the progression of injury and favor tissue regeneration.^11^, ^12^ Moreover, many other cell types, either resident (cholangiocytes lining in the biliary duct, cells building the lymphatic vessels, portal fibroblasts, major vessels endothelial cells, liver progenitor stem cells) or transient (immune cells such as T and B lymphocytes, natural killer cells, neutrophils or erythrocytes), are found in the liver constituting the remaining 5% of its mass.^1^, ^13^, ^14^, ^15^, ^16^

The key role of NPC in liver (patho)physiology is getting more notorious. Hence, isolation of primary cells from pre‐clinical experimental models is crucial. However, and especially in the field of chronic liver disease (CLD), few reports have described detailed protocols for simultaneous isolation of the main hepatic cell types from cirrhotic rodent models.^17^

In 1972, Seglen^18^ achieved an important advance by introducing the two‐step perfusion technique for isolating rat liver cells. It was not until 2011 when Liu et al^19^ described a detailed systematic method for simultaneous isolation of hepatocytes and NPC by means of low‐speed centrifugations. Nevertheless, several alternative techniques have been developed to isolate NPC. The most extended being density gradient centrifugation,^20^, ^21^ counterflow elutriation,^5^, ^22^ fluorescence activated cell sorting (FACS)^17^ and immunomagnetic bead isolation also known as MACS.^23^

Cell density separation is highly useful to isolate HSC from the NPC suspension. Generally, the densities of LSEC and HMΦ overlap; therefore, further steps are required to purify both cell types. For example, short incubation of these cells on non‐coated substrates is sufficient for HMΦ to attach while LSEC can be easily recovered from the suspension.^24^, ^25^ Counterflow elutriation has been intensively used for obtaining highly pure LSEC, although it is time‐consuming, expensive and requires specialized equipment. The use of FACS and MACS as the gold standard techniques for liver cell isolation has been reasonably questioned as the overall yield is generally low and reliable surface markers are doubtfully available for liver sinusoidal cell populations. Indeed, isolation methods based on “specific” antibody labelling against membrane receptors have two main limitations, especially in disease models: antibody‐receptor recognition and binding could trigger intracellular signaling further altering the naïve properties of the isolated cells, and the expression of surface markers may substantially vary due to disease‐mediated changes in cell phenotype, thus a particular cell population may be exclusively selected, excluding cells from the same community with slightly different phenotype.

For all the technical requirements and controversies regarding NPC phenotype, the isolation of liver cells based on their characteristics with regard to density and substrate adherence time remain the primary method used to separate these cell populations. Consequently, the aim of this work was to develop a fast, cheap and user‐friendly reproducible protocol for the simultaneous isolation of the four main liver cell types from a single rat liver without antibody selection and suitable for different models of CLD. Isolated cells obtained with the herein presented protocol were tested for purity, yield and functionality.

## 2 MATERIALS AND METHODS

### 2.1 Animals

Wistar Han rats weighting 300‐350 g were used as healthy (control: Ct) animals. Liver cirrhosis (Ch) was induced in 50‐75 g Wistar Han rats by chronic inhalation of carbon tetrachloride (Ch‐CCl_4_) three times a week and receiving 0.3 g/L phenobarbital in the drinking water. When rats developed ascites, approximately after 14‐16 weeks, toxicants administration was stopped. For thioacetamide‐induced cirrhotic animals (Ch‐TAA), TAA was dissolved in 0.9% saline approximately 4 h before injection. Cirrhosis induction was evident after 12 weeks of treatment (250 mg/kg of TAA twice a week). Control and cirrhotic animals (n = 6 per experimental group) were weight and age matched.

Animals were kept at the University of Barcelona Faculty of Medicine facilities with controlled temperature (19.7 ± 2°C), humidity (52 ± 5%) and light/dark cycle (12 hours each). Animals were fed ad libitum with water and standard rodent food pellets and housed in conventional cages. All experiments were approved by the Laboratory Animal Care and Use Committee of the University of Barcelona and were conducted in accordance with the European Community guidelines for the protection of animals used for experimental and other scientific purposes (European Economic Community (EEC) Directive 86/609). All animals were supplied by Charles River Laboratories International, Inc.

### 2.2 Liver perfusion and digestion

**Figure 1:**
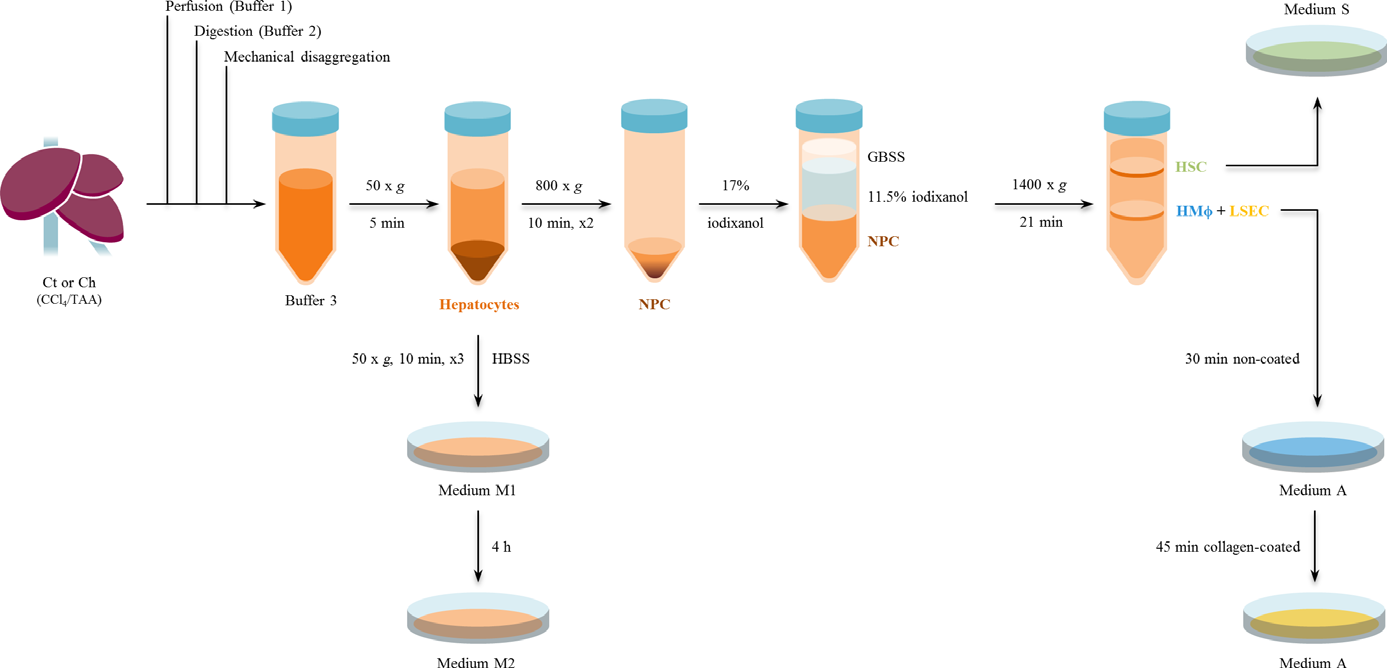
Summarized overview of hepatic cells isolation procedure. Control and cirrhotic livers (n = 6) were perfused, digested with collagenase A, and mechanically disaggregated obtaining a multicellular suspension. Hepatocytes were purified by low revolution centrifugations. Non‐parenchymal cells were subjected to a differential centrifugation using iodixanol obtaining a pure fraction of HSC and another one of mixed LSEC+HMΦ. Due to the ability of LSEC to specifically adhere to coated substrates, we were able to obtain highly enriched LSEC and HMΦ cultures

Table 1 describes the different buffers required for the protocol. Rats were intraperitoneally anesthetized with a combination of 100 mg/kg ketamine and 5 mg/kg midazolam and sprayed with 96% ethanol to set an aseptic environment. A mid‐abdominal incision towards the sternum was made and the intestines were displaced to expose the portal vein. 500 μL heparin were injected through the cava vein and the liver was perfused (20G catheter) through the portal vein with pre‐warmed Buffer 1 for 10 minutes at a flow rate of 20 mL/min. Simultaneously, the cava vein was cut to allow outflow of the solution. After perfusion, the liver was digested with pre‐warmed Buffer 2 for 30 minutes at a flow rate of 5 mL/min. The resultant digested liver was excised, cut up and in vitro digestion were performed with Buffer 2 supplemented with 0.01% collagenase A (30% extra collagenase A was added for cirrhotic livers). Disaggregated tissue was filtered using a 100 μm nylon strainer, collected in cold Buffer 3 and centrifuged at 50× *g* for 5 minutes. The pellet contained hepatocytes, while NPC were found in the supernatant. Complete protocol for further purification of liver cells is detailed in the following paragraphs and summarized in Figure 1.

**Table 1:** Buffers composition. Detailed reagents for the preparation of perfusion, digestion and suspension buffers

Reagent	Concentration			Product reference
	Buffer 1	Buffer 2	Buffer 3	
Hepes	12 mmol L^−1^	12 mmol L^−1^	25 mmol L^−1^	H3375, Sigma, Barcelona, Spain
Ethylene glycol‐bis (2‐aminoethylether)‐N,N,N′,N′‐tetraacetic acid (EGTA)	0.6 mmol L^−1^	‐	‐	E4378, Sigma
Bovine serum albumin (BSA)	0.23 mmol L^−1^	‐	‐	A1391.0100, Panreac Applichem, Barcelona, Spain
Sodium bicarbonate (NaHCO_3_)	25 mmol L^−1^	25 mmol L^−1^	0.0025 mol L^−1^	S6297, Sigma
Sodium chloride (NaCl)	0.14 mol L^−1^	0.14 mol L^−1^	0.125 mmol L^−1^	S9625, Sigma
Potassium chloride (KCl)	0.005 mol L^−1^	0.005 mol L^−1^	0.005 mol L^−1^	P9333, Sigma
Magnesium sulfate heptahydrate (MgSO_4_·7H_2_O)	0.001 mol L^−1^	0.001 mol L^−1^	1.75 mmol L^−1^	230391, Sigma
di‐Sodium hydrogen phosphate dihydrate (Na_2_HPO_4_·2H_2_O)	0.3 mmol L^−1^	0.3 mmol L^−1^	‐	6580.0500, Merck, Darmstadt, Germany
Potassium phosphate monobasic (KH_2_PO_4_)	0.4 mmol L^−1^	0.4 mmol L^−1^	1 mmol L^−1^	P5379, Sigma
Heparin (1000 IU)	1%	‐	‐	34102, Rovi, Barcelona, Spain
Calcium chloride dihydrate (CaCl_2_·2H_2_O)	‐	4 mmol L^−1^	2.5 mmol L^−1^	C3881, Sigma
Collagenase A	‐	0.015%	‐	103586, Roche, Barcelona, Spain

All buffers were set at physiological pH 7.4 and oxygenated (95% O_2_ + 5% CO_2_) for 20 minutes.

### 2.3 Isolation of primary rat hepatocytes

Pellet containing hepatocytes was rinsed with cold Hanks’ balanced salt solution (HBSS) and subsequently centrifuged (50× *g*, 5 min, 4°C). This washing procedure was performed three times. Hepatocytes above 75% viability (evaluated by trypan blue exclusion) were cultured in medium M1 (Table 2) plated in 0.1 mg/mL collagen‐coated petri dishes at a density of 109 000 cells/cm^2^ and maintained at 37°C in a humidified atmosphere of 5% CO_2_. After 4 hours, cells were rinsed twice with Dulbecco's phosphate‐buffered saline (DPBS) and the medium was replaced by medium M2 (Table 2).

**Table 2:** Medium composition. Detailed supplements and concentrations for cell culture media

Reagent	Concentration				Product reference
	Medium M.1	Medium M.2	Medium A	Medium S	
Dulbecco's Modified Eagle's Medium (DMEMF12)	✓	✓	‐	‐	11320074, Gibco, Madrid, Spain
Roswell Park Memorial Institute medium 1640 without L‐glutamine (RPMI)	‐	‐	✓	‐	01‐101‐1A, Biological Industries, Cromwell, CT, USA
Iscove's Modified Dulbecco's Medium (IMDM)	‐	‐		✓	21980‐032, Invitrogen, Madrid, Spain
Fetal Bovine Serum (FBS)	10%	2%	10%	10%	04‐001‐1A, Biological Industries
Penicillin plus streptomycin	1%	1%	1%	1%	03‐331‐1C, Biological Industries
L‐glutamine	2 mmol L^−1^	2 mmol L^−1^	2 mmol L^−1^	2 mmol L^−1^	25030‐024, Gibco
Amphotericin B^a^	1%	1%	1%	1%	03‐029‐1C, Biological Industries
Dexamethasone^a^	1 μmol L^−1^	1 nmol L^−1^	‐	‐	D4902, Sigma
Insulin^a^	1 μmol L^−1^	1 μmol L^−1^	‐	‐	Humulin S, Lilly S.A.
Endothelial cell growth supplement (ECGS)^a^	‐	‐	50 μg/mL	‐	BT‐203, Alfa Aesar, Karlsruhe, Germany
Heparin^a^	‐	‐	100 μg/mL	‐	H3393, Sigma

a Freshly added before cell seeding.

### 2.4 Purification of primary non‐parenchymal cells

The supernatant containing NPC was centrifuged to eliminate remaining hepatocytes (50× *g* for 5 minutes at 4°C) and the supernatant was centrifuged twice (800× *g* for 10 minutes at 4°C) to wash and precipitate the NPC. The obtained pellet was resuspended in 15 mL of 17% iodixanol diluted in Gey's balanced salt solution (GBSS). Three 15 mL tubes were filled with 5 mL of the multicellular suspension and 5 mL of 11.5% iodixanol were carefully overlaid onto the cell suspension followed by 2 mL of GBSS. After centrifugation at 1400× *g* for 21 minutes at 4°C without break, two interphases were obtained; the lower interphase contained HMΦ and LSEC while the upper interphase was enriched in HSC.

### 2.5 Isolation of hepatic macrophages and liver sinusoidal endothelial cells

HMΦ and LSEC‐enriched fraction was carefully collected, diluted in DPBS and centrifuged at 800× *g* for 10 minutes at 4°C. The cell pellet was resuspended in medium A (Table 2), seeded on non‐coated petri dishes and incubated for 30 minutes at 37°C in humid atmosphere with 5% CO_2_ in order to enhance LSEC purity by selective adherence time of HMΦ. Non‐adhered cells (LSEC fraction) were seeded on collagen‐coated substrates and maintained for 45 minutes at the previous incubation conditions. Afterwards, cells were washed twice with DPBS and left overnight (O/N) (37°C, 5% CO_2_) in medium A.

### 2.6 Isolation of hepatic stellate cells

HSC‐enriched interphase was carefully collected and rinsed with GBSS. After centrifugation at 800× *g* for 10 minutes at 4°C the cell pellet was resuspended in medium S (Table 2) and plated on non‐coated petri dishes. HSC were maintained at 37°C in a humidified atmosphere of 5% CO_2_ O/N.

### 2.7 Cell yield and viability

Yield and viability of each cell type were evaluated in Ct and cirrhotic animals (Ch‐CCl_4_ and Ch‐TAA) by trypan blue exclusion assessed by two independent researchers.

Yield per gram of tissue was calculated considering liver weight averages of 9, 10 and 13 g for Ct, Ch‐CCl_4_ and Ch‐TAA respectively. Functional characterization was performed in cells isolated from Ct and Ch‐CCl_4_ rats.

### 2.8 Immunocytofluorescence

Isolated cells were cultured in petri dishes and fixed with 4% paraformaldehyde for 10 minutes, rinsed three times with DPBS and permeabilized for 5 minutes with 0.1% triton. After rinsing 3 times with DPBS, cells were blocked for 30 minutes. Fixed cells were incubated with cell type specific primary antibody: 1/63 albumin (MAB1455, R&D Systems, Minneapolis, MN, USA) for hepatocytes, 1/100 rat endothelial cell antigen 1 (Reca‐1) (MCA970R, Biorad, Madrid, Spain) for LSEC, 1/100 cluster of differentiation 68 (CD68) (MCA341R, Biorad) for HMΦ and 1/100 desmin (M0760, Dako, Madrid, Spain) for HSC. After 45 minutes, cells were incubated with 1/300 Alexa Fluor 488‐conjugated donkey antimouse secondary antibody (A‐21202, Thermo Fisher Scientific, Madrid, Spain) and 1/1000 Hoechst (D1306, Thermo Fisher Scientific) for 1 hour. Finally, coverslips were placed onto cells with fluoromount‐G medium. Blocking, primary antibody and secondary antibody solutions were prepared with 1% Bovine Serum Albumin dissolved in DPBS and incubated at room temperature.

Immunocytofluorescence staining was examined using a fluorescence microscope (Olympus BX51, Tokyo, Japan) equipped with a digital camera (Olympus, DP72). Five representative images were taken from each preparation at 200× magnification. Image analysis was performed with Fiji‐ImageJ (National Institutes of Health, Bethesda, MD, USA). For each cell type, more than 700 cells were analysed. Purity of each cell culture was calculated as the number of positive cells (for their type‐specific marker) divided by the number of Hoechst‐positive cells. Negative controls included the incubation of each cell type with antibodies specific for the other hepatic cells sub‐populations. Images were counted by two independent researchers blindly.

### 2.9 Albumin and urea production

Supernatant from healthy and cirrhotic hepatocytes cultured at different densities (2.5 × 10^5^ and 5 × 10^5^ cells/well) was collected after 24 hours of culture. Albumin and urea nitrogen (BUN) were measured using standard methods at the Hospital Clínic of Barcelona's CORE laboratory. BUN values were converted to urea as 2.1428 mg/dL BUN = 1 mg/dL urea.

### 2.10 Acetylated low‐density lipoprotein assay

The endocytic capacity of LSEC was assessed by acetylated low‐density lipoprotein (Ac‐LDL) uptake. In this regard, LSEC cultured for 12 hours upon isolation were rinsed twice with pre‐warmed DPBS, incubated with 5 μg/mL Alexa Fluor 488 Ac‐LDL (L23380, Invitrogen) and 1 μmol L^−1^ Hoechst diluted in medium A without phenol red for 30 minutes at 37°C protected from light. Then, cells were rinsed with DPBS and fresh media was added for further assessment in the microscope. Six images of each sample (200× magnification) were analysed.

### 2.11 Scanning electron microscopy

LSEC fenestration was assessed by scanning electron microscopy (SEM). LSEC were fixed O/N with 2% glutaraldehyde dissolved in 0.1 mol L^−1^ cacodylate buffer pH 7.4 for 30 minutes at room temperature, washed with cacodylate buffer three times for 5 minutes and incubated with tannic acid (1%) for 1 hour followed by 2 hours incubation with 2% osmic acid. Samples were dehydrated with an ethanol battery (50%, 70%, 90%, 95% and 100%), critical‐point dried with hexamethyldisilazane, sputter‐coated with gold and examined by SEM.

### 2.12 HMΦ response to lipopolysaccharide stimulation

Isolated HMΦ were incubated with 100 ng/mL lipopolysaccharide (LPS) from *Escherichia coli* (L2630, Sigma) or its vehicle (DPBS) for 6 hours. Then, cells were rinsed and properly preserved at −80°C with RLT buffer (Qiagen, Madrid, Spain) containing 10 mmol L^−1^ β‐mercaptoethanol for further analysis of mRNA expression: mannose receptor C type 1 (Mrc1), arginase 1 (Arg1) and interleukin‐10 (IL‐10) as anti‐inflammatory mediators, CCL2, also known as MCP1, interleukin‐1β (IL‐1β), interleukin‐6 (IL‐6) and inducible nitric oxide synthase (iNOS) as pro‐inflammatory signals and TNFα considered as modulator of immune response.

### 2.13 RT‐PCR

mRNA was isolated and purified using RNeasy Mini Kit (74104, Qiagen) according to manufacturer's instructions. RNA was quantified using Nanodrop software (ND1000, Marshall Scientific, Hampton, NH, USA) and reverse transcribed to cDNA using Quantitect Reverse Transcription Kit (205311, Qiagen) previous elimination of genomic DNA of the sample. cDNA templates were amplified by real‐time TaqMan polymerase chain reaction (Taqman Fast Universal PCR Master Mix, 4352042, Applied Biosystems, Madrid, Spain) on an ABI ;Prism 7900HT Fast Detection System (Applied Biosystems). Expression of Mrc1 (Rn01487 342_m1), Arg1 (Rn00691090_m1), IL‐10 (Rn00563409_m1), TNFα (Rn01525859_g1), CCL2 (Rn00580555_m1), IL1β (Rn00580432_m1), IL‐6 (Rn01410330_m1) and iNOS (Rn00561646_m1) were analysed using predesigned gene expression assays from Applied Biosystems (Thermo Fisher Scientific). CT values were normalized to those of GAPDH (Rn01775763_g1) and expressed as relative changes vs the control (ΔΔCT method). All PCR reactions were performed in duplicate and using nuclease‐free water as controls.

### 2.14 HSC in vitro activation

Isolated HSC were activated in vitro with consecutive trypsinization. Once a week, HSC cultured in 25 cm^2^ flask were trypsinized using 0.05% EDTA‐trypsin until passage 4. HSC protein lysate was obtained in every trypsinization passage. Medium S was replaced thrice a week.

### 2.15 Western Blotting

HSC protein lysates were run on a SDS‐polyacrylamide gel and transferred to a nitrocellulose membrane. Blots were blocked for 1 hour and probed O/N at 4°C with antibodies (diluted 1/1000) against alpha smooth muscle actin (α‐SMA) (A2547, Sigma) or GAPDH (SC‐32233, Santa Cruz, CA, USA) as a housekeeping gene. Chemiluminescence analysis of the blots was performed using Image Studio (LI‐COR Biosciences, Lincoln, NE, USA).

### 2.16 Chemicals

For liver cirrhosis induction, CCl_4_ (289116, Sigma) and phenobarbital (Kern Pharma, Barcelona, Spain) or TAA (172502, Sigma) were used. Rats were anesthetized with ketamine (448.00.03, Merial, Barcelona, Spain) and midazolam (841155.9, Normon SA, Madrid, Spain). Collagenase A (10103586001, Roche) was used for tissue digestion. Reagents for cell isolation included HBSS (H8264, Sigma), collagen type 1 rat tail (A10483‐01, GIBCO), DPBS (L0615‐500, Biowest, Barcelona, Spain), iodixanol (D1556, Sigma) and GBSS (G9779, Sigma). For immunocytofluorescence identification, paraformaldehyde (sc‐281694, SantaCruz Biotechnology, Madrid, Spain), triton (×100, Sigma) and fluoromount‐G medium (100‐01, Southern Biotech, Birmingham, AL, USA) were used. SEM preparations required glutaraldehyde (G5882, Sigma) and HMDS (440191, Sigma).

### 2.17 Statistical analysis

Statistical analysis was performed with SPSS Statistics 19 (Armonk, NY, USA) software for Windows. Results were expressed as mean ± standard error of mean. In order to assess differences between‐groups we performed Student's *T*‐test when variables were parametric and Mann‐Whitney test for non‐parametric variables. Differences between groups were considered as significant when *P*‐value ≤0.05.

## 3 RESULTS

### 3.1 Hepatic cells isolation: Yield and purity

The viability and yield of hepatic cells isolated from Ct and Ch rats are shown in Table 3. Ct livers yielded more hepatocytes than Ch ones, showing viability around 80%. Isolation of NPC resulted in similar yields of LSEC in all groups, but as expected, the yield of HSC isolated from Ch livers was significantly greater than Ct. HMΦ seemed to be reduced in cirrhotic livers, although this difference was not significant. Viability of NPC in both Ct and Ch rats was ≥95%. No differences in the amount of each type of cells were observed comparing both pre‐clinical models of cirrhosis.

**Table 3:** Yield and purity of isolated hepatic cells

	Hepatocytes			LSEC			HSC			HMΦ		
	Ct	Ch‐CCl_4_	Ch‐TAA	Ct	Ch‐CCl_4_	Ch‐TAA	Ct	Ch‐CCl_4_	Ch‐TAA	Ct	Ch‐CCl_4_	Ch‐TAA
Yield (cells/g tissue)^a^	3 ± 0.4 × 10^7^	**9.5 ± 1.8 × 10** ^**6**^	**8.4** ± **1.2 × 10** ^**6**^	2.2 ± 0.7 × 10^6^	4.7 ± 1.8 × 10^6^	2.5 ± 0.6 × 10^6^	6.7 ± 1.9 × 10^5^	**1.2** ± **0.2 × 10** ^**6**^	**1.3** ± **0.3 × 10** ^**6**^	3.6 ± 1.3 × 10^6^	1.8 ± 0.8 × 10^6^	1.2 ± 0.4 × 10^6^
Viability (%)^b^	77 ± 3	80 ± 2	80 ± 3	96 ± 1	97 ± 1	96 ± 1	87 ± 2	93 ± 2	89 ± 2	95 ± 1	92 ± 6	93 ± 1
Purity (%)^c^	96 ± 1	95 ± 1	‐	98 ± 1	96 ± 2	‐	96 ± 1	95 ± 2	‐	98 ± 1	95 ± 3	‐

Values in bold represent significant differences vs Ct‐cells (*P*‐value ≤ 0.05). Each value represents mean ± standard error of the mean for 6 independent hepatic isolations.

a Yield was calculated as total cells per gram of tissue in Ct, Ch‐CCl_4_ and Ch‐TAA livers weighting 9, 10 and 13 g respectively.

b Viability was analyzed by trypan blue exclusion.

c Purity was determined by immunofluorescence staining of specific proteins: albumin (hepatocyte), Reca‐1 (LSEC), desmin (HSC) and CD68 (HMΦ).

LSEC, liver sinusoidal endothelial cells; HSC, hepatic stellate cells; HMΦ, hepatic macrophages; Ct, control; Ch, cirrhotic; TAA, thioacetamide.

**Figure 2:**
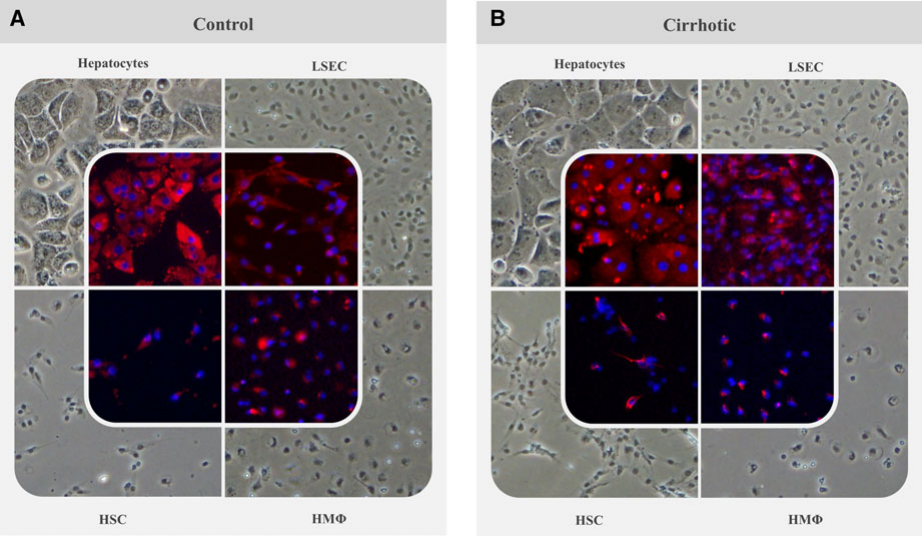
Immunofluorescence characterization of isolated cells. Phase‐contrast images of primary isolated hepatic cells were taken showing characteristic morphology of control (A) and cirrhotic (B) sinusoidal cells in vitro. Further immunofluorescent characterization was performed with specific markers for each cell type: albumin (hepatocytes), Reca‐1 (LSEC), desmin (HSC) and CD68 (HMΦ). Original images of these preparations are included in Figure S1

Hepatic cells were morphologically characterized in phase‐contrast images (Figure 2 and Figure S1). Hepatocytes showed their characteristic mono(bi)‐nucleated polygonal shape, freshly isolated LSEC were small and round, HSC exhibited lipid droplets and characteristic stellate shape, and HMΦ are recognized by their stellate‐macrophage shape. Additionally, identity and purity of isolated hepatocytes and NPC were evaluated by immunofluorescence of specific proteins for each cell type: albumin (hepatocytes), Reca‐1 (LSEC), desmin (HSC) and CD68 (HMΦ) (Figure 2). As shown in Table 3, purity of Ct or Ch hepatic cells isolation was ≥95%. Importantly, no fluorescence signal was obtained when each hepatic cell sub‐population was incubated with specific antibodies for the other cell types and analyzed using the same experimental parameters (data not shown).

### 3.2 Functional characterization of isolated hepatic cells

#### 3.2.1 Hepatocytes

**Figure 3:**
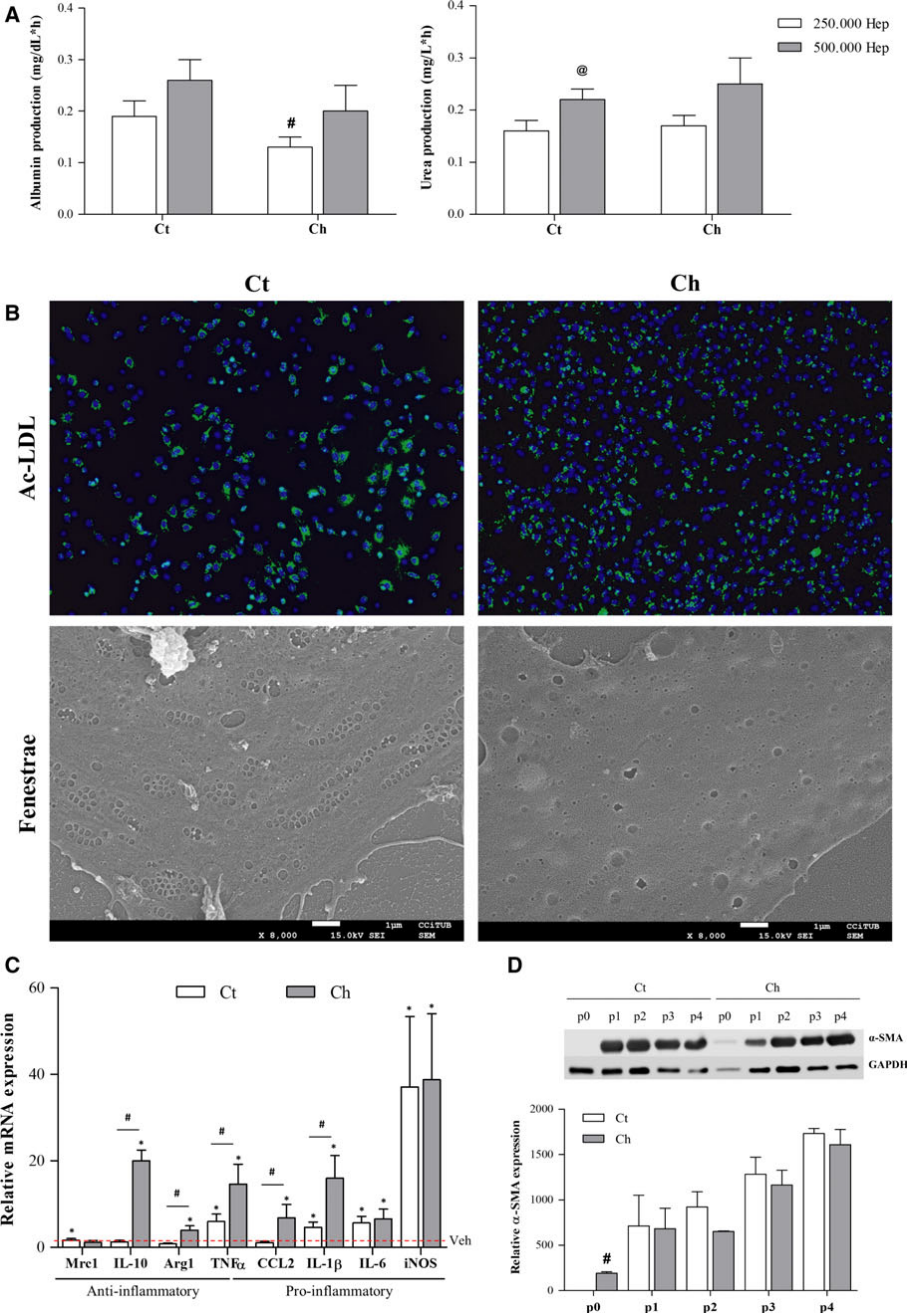
Functional characterization of isolated cells. A, Hepatocytes functionality was evaluated as albumin (left) and urea (right) release to the culture media. B, LSEC were assessed for their endocytic ability to incorporate fluorescent Ac‐LDL (top) (green) and fenestrae presence (bottom) evaluated by SEM. C, mRNA expression of the anti‐inflammatory genes Mrc1, IL‐10 and Arg1, the pro‐inflammatory genes CCL2, IL‐1β, IL‐6 and iNOS, and TNFα were evaluated in Ct and Ch HMΦ in response to LPS. Red dotted line represents each vehicle‐treated group. D, Representative Western Blot and corresponding quantification of α‐SMA evaluated in HSC isolated form Ct and Ch rat livers during in vitro culture. Significant differences: ^#^ *P*‐value ≤0.05 vs Ct; ^@^ *P*‐value ≤0.05 vs 250.000; *P‐value ≤0.05 vs Veh

Primary hepatocytes isolated from Ct or Ch rats were cultured in 6‐well plates at a density of 2.5 × 10^5^ and 5 × 10^5^ viable cells/well. After 24 hours of culture, cell supernatant was collected for subsequent analysis. Both Ct and Ch hepatocytes synthesized and released albumin and urea to the culture media, which was dependent on cell density, thus demonstrating cell functionality. In addition, albumin production from Ch hepatocytes was lower than that of Ct cells, although this difference was not statistically significant (Figure 3A).

#### 3.2.2 Liver sinusoidal endothelial cells

Functionality of primary isolated LSEC was analyzed in terms of their endocytic capacity and fenestrae presence. As shown in Figure 3B, LSEC isolated from Ct or Ch rats were able to uptake the fluorescent Ac‐LDL. In addition, the endothelium transmembrane perforations, or fenestrae, characteristic of differentiated LSEC were evaluated by SEM. As expected, Ct‐LSEC exhibited numerous fenestrae grouped in sieve plates whereas Ch‐LSEC showed a typical de‐differentiated phenotype with reduced number and diameter of fenestrae (Figure 3B).

#### 3.2.3 Hepatic macrophages

Hepatic macrophages, as cells of the immune system, are able to respond to different pro‐inflammatory stimuli. As shown in Figure 3C, HMΦ isolated from Ct animals increased the expression of most pro‐inflammatory markers (TNFα, IL‐1β, IL‐6 and iNOS) in response to LPS without changes in anti‐inflammatory markers other than Mrc1. Regarding cirrhotic animals, all the analyzed markers except Mrc1 were significantly increased in response to LPS. Furthermore, HMΦ isolated from cirrhotic animals exhibited an exacerbated response to LPS when compared to HMΦ isolated from healthy individuals as shown by significant increases in Arg1, IL‐10, TNFα, CCL2 and IL‐1β.

#### 3.2.4 Hepatic stellate cells

Assessment of cell activation during in vitro culture was used as marker of HSC functionality. Figure 3D shows the increase in the activation marker α‐SMA in Ct and Ch HSC upon trypsinization and during in vitro culture. When comparing Ct vs Ch, basal expression of α‐SMA (p0) was significantly increased in cirrhotic animals. Nevertheless, no significant differences were seen in the subsequent over‐activated cells (activation passages p1 to p4).

## 4 DISCUSSION

In order to evaluate the pathophysiology or novel treatments for liver diseases, in vivo models have several advantages over cell cultures. For example, in vivo, the different hepatic cell types are kept in their 3D physiological microenvironment, with proper direct contact or paracrine communication with other cell types and are influenced by systemic blood components and the immune system.^3^ However, in vitro cultures also feature several advantages over in vivo models; they allow the assessment of drugs’ direct effects on the different cell types or facilitate the study of specific cell communication (cross‐talk) in response to a therapeutic agent.^26^ In addition, in vitro models have ethical advantages as they reduce or replace otherwise required experimental animals.

For these and additional reasons, it is generally accepted that a combination of both tools is required in pre‐clinical translational research. In this regard, multi‐cell in vitro liver cultures such as liver‐on‐a‐chip devices,^27^ organoids^28^ or precision cut liver slices^29^, ^30^ are becoming popular as they combine the versatility of in vitro cultures while partially simulating the in vivo microenvironment, showing improved function and drug‐response over conventional cultures.

Although freshly isolated liver cells would be the optimal choice for the above mentioned in vitro models (either for conventional cultures or multi‐cell systems), the difficulties and expenses associated with cellular isolation can drive researchers to other alternatives such as the use of immortalized cell lines or primary in vitro‐expanded cultures, which may have lost the highly specialized features found in liver cells in vivo.^31^, ^32^, ^33^

In this manuscript, we describe and characterize a novel, easy and affordable protocol for the isolation of the four main hepatic cell types from a single rat liver. Importantly, we clearly detail the entire method, and release the complete list of reagents and solutions to allow a proper replication by any researcher from any institution.

One of the advantages of this protocol lies on its independence on antibody‐mediated selection, thus avoiding concerns about undesired activation of membrane receptors and subsequent molecular pathways (positive selection) or unwanted enrichment of a cellular subpopulation (positive/negative selection). Instead, the protocol is based mainly in fractionation by centrifugation. In this regard, although loss of sample or inter‐fraction contamination would be expected of any standard centrifugation protocol, the results show good yields for each cell type (in the order of millions) and the repeated wash‐centrifugation cycles ensure a purity above 95% in all cases.

In addition to healthy livers, we herein validated the protocol in livers from two different models of CLD. It is well known that cirrhotic rats (either CCl_4_‐ or TAA‐induced) display high amount of ECM,^30^, ^34^ which in combination with over‐constriction of sinusoids causes an increase in the intra‐hepatic vascular resistance (primary cause in the development of portal hypertension).^4^, ^35^ Despite the fact that our protocol relies on perfusion of the liver in order to accomplish a proper digestion, we herein show that this protocol is suitable not only for healthy livers (with perfectly arranged sinusoids) but also in models of cirrhosis (distorted hepatic architecture and microcirculation). Interestingly, yields reproduced the expected changes in cell population during cirrhosis, with a significant reduction on hepatocytes (probably due to described parenchymal extinction^36^), a greater number of HSC^12^ and reduced HMΦ infiltration.^37^ Regardless, the order of magnitude of the yield for all cell types remained similar, ensuring comparable amounts of starting sample for any experiment to that of control isolations.

As described above, one of the main advantages of freshly isolated liver cells over cell lines is the preservation of their specific phenotype (at least for the first hours in vitro). That is why we validated the phenotype of the four cell types obtained after this procedure, both with molecular markers and functional assays to determine their ability to respond to stimuli. Indeed, hepatocytes produced albumin and urea,^38^ LSEC displayed characteristic fenestrae and sinusoidal Ac‐LDL endocytosis,^39^, ^40^, ^41^ HMΦ responded to LPS stimulation, especially those isolated from Ch animals, which is in accordance with recent in vivo data on acute on chronic liver failure,^34^ and HSC expressed their characteristic activation marker α‐SMA. In addition, cells isolated from cirrhotic livers exhibited a phenotype that matched their pathologic situation (hepatocytes: reduced albumin synthesis,^42^ LSEC: lack of fenestrae,^43^ HMΦ: inflammatory hyperesponse,^44^ HSC: increased α‐SMA expression^45^) suggesting that, unlike other protocols where selection is based on surface markers that may change during cirrhosis (CD31, CD32b)^33^ our protocol is phenotype‐unbiased and thus suitable for the study of different models of CLD. For this same reason, we do not discard the applicability of the 4 in 1 protocol in other models of liver diseases, such as non‐alcoholic steatohepatitis or warm ischemia/reperfusion, although further studies are required in this matter.

Finally, the distinctive characteristic of this protocol is that it allows the isolation of the main hepatic cells from just one animal. This has several implications that represent an advantage to other existing protocols and, in fact, address current needs of the scientific community:

1. Reduction of the number of animals used (ethical improvement) while the required materials are essentially the same to that of a single cell type isolation (economic improvement).
2. Possibility of performing cross‐talk experiments or assembling multicellular in vitro culture systems with cells from the same animal (and thus with certainty that they have the same degree of activation/damage or stage of the disease).
3. The 4 in 1 protocol may be used to isolate cells from an animal that has been treated in vivo with a certain therapeutic, thus allowing separate response analysis for each cell type and correlation with in vivo data from the animal (AST, ALT, hemodynamic data among others).

Indeed, only few studies have reported successful separation of the four cell types at once.^19^, ^20^, ^21^, ^22^ Comparison of yields and purity with previous bibliography in the field shows similar results to those obtained using the herein proposed protocol. However, the published protocols rely on antibody fractionation using FACS or MACS (with the abovementioned associated limitations), require multiple gradient centrifugation steps (thus with greater duration and cost), and/or rely on the use of pronase, which may be detrimental for hepatocyte yield and viability. Moreover, none of them assessed the suitability for isolation of cells from diseased livers.

In conclusion, we herein describe an antibody‐free protocol for simultaneous isolation of hepatocytes, LSEC, HSC and HMΦ with optimal yield, purity and viability, which is suitable for healthy or cirrhotic livers. Its low equipment requirements and quick feasibility make it suitable for laboratories with standard cell‐culture facilities, thus encouraging the use of freshly isolated primary cells over sub‐optimal in vitro models.

## AUTHORS’ CONTRIBUTIONS

A.F.‐I. and M.O.‐R. conceived the study, designed the research, performed experiments, analysed data and wrote the manuscript. S.G.‐M. critically analyzed data and wrote the manuscript. J.G.‐S. conceived the study, designed and directed the research, wrote the manuscript and obtained funding. All authors edited and reviewed the final manuscript.

## CONFLICT OF INTEREST

The authors of this manuscript have no conflicts of interest to disclose as described by the Journal of Cellular and Molecular Medicine.
