# Supplementary figures and images for: 4 in 1: Antibody‐free protocol for isolating the main hepatic cells from healthy and cirrhotic single rat livers
Source: J Cell Mol Med. 2018 Nov 12;23(2):877–86. doi: 10.1111/jcmm.13988 (PMC6349241; doi:10.1111/jcmm.13988)

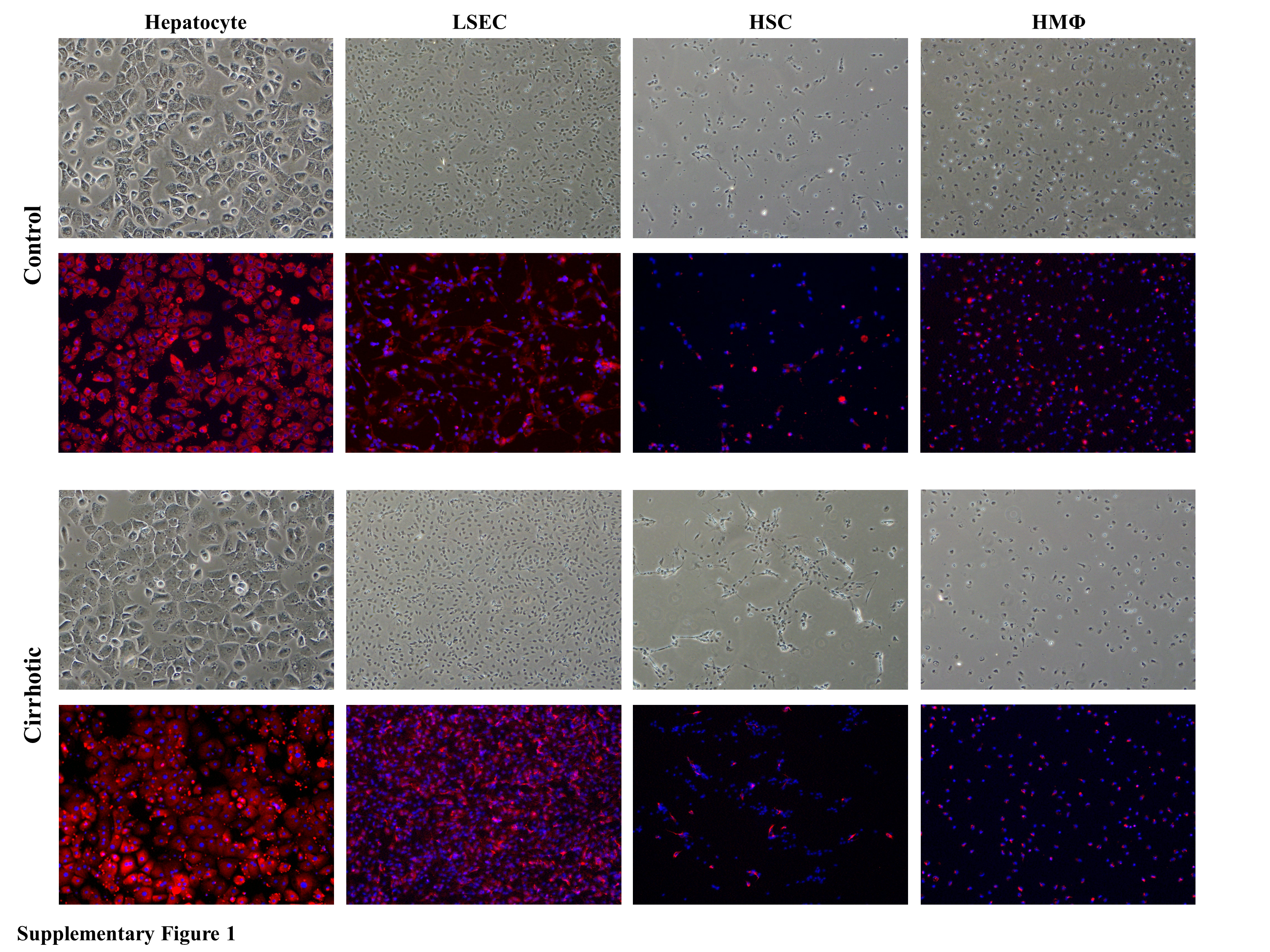

Supplement: Supplementary file 1 [file JCMM-23-877-s001.TIF]
