# Supplementary material for: 4 in 1: Antibody‐free protocol for isolating the main hepatic cells from healthy and cirrhotic single rat livers
Source: J Cell Mol Med. 2018 Nov 12;23(2):877–86. doi: 10.1111/jcmm.13988 (PMC6349241; doi:10.1111/jcmm.13988)
Supplement: Supplementary file 2 [file JCMM-23-877-s002.docx]

**4 in 1: Antibody-free protocol for isolating the main hepatic cells from healthy and cirrhotic single rat livers**

Anabel Fernández-Iglesias, Martí Ortega-Ribera, Sergi Guixé-Muntet,

Jordi Gracia-Sancho

**Supplementary figure legend**

**Supplementary Figure 1. Immunofluorescence characterization of isolated cells.** Phase-contrast images of primary isolated hepatic cells were taken showing characteristic morphology of control (A) and cirrhotic (B) sinusoidal cells *in vitro*. Further immunofluorescent characterization was performed with specific markers for each cell type: albumin (hepatocytes), Reca-1 (LSEC), desmin (HSC) and CD68 (HMΦ). Five representative images at 200x magnification were taken for each preparation.
